# Increased standardized incidence ratio of breast cancer in female electronics workers

**DOI:** 10.1186/1471-2458-7-102

**Published:** 2007-06-08

**Authors:** Tzu-I Sung, Pau-Chung Chen, Lukas Jyuhn-Hsiarn Lee, Yi-Ping Lin, Gong-Yih Hsieh, Jung-Der Wang

**Affiliations:** 1 Institute of Occupational Medicine and Industrial Hygiene, National Taiwan University College of Public Health, No. 17 Xuzhou Road, Taipei 100, Taiwan; 2 Center for Health Risk Assessment and Policy, National Taiwan University College of Public Health, No. 17 Xuzhou Road, Taipei 100, Taiwan; 3 Department of Neurology, National Taiwan University Hospital, Taipei, Taiwan; 4 Department of Public Health, National Taiwan University College of Public Health, Taipei, Taiwan; 5 Department of Internal Medicine and Department of Environmental and Occupational Medicine, National Taiwan University Hospital, No.1 Changde Street, Taipei 100, Taiwan

## Abstract

**Background:**

In 1994, a hazardous waste site, polluted by the dumping of solvents from a former electronics factory, was discovered in Taoyuan, Taiwan. This subsequently emerged as a serious case of contamination through chlorinated hydrocarbons with suspected occupational cancer. The objective of this study was to determine if there was any increased risk of breast cancer among female workers in a 23-year follow-up period.

**Methods:**

A total of 63,982 female workers were retrospectively recruited from the database of the Bureau of Labor Insurance (BLI) covering the period 1973–1997; the data were then linked with data, up to 2001, from the National Cancer Registry at the Taiwanese Department of Health, from which standardized incidence ratios (SIRs) for different types of cancer were calculated as compared to the general population.

**Results:**

There were a total of 286 cases of breast cancer, and after adjustment for calendar year and age, the SIR was close to 1. When stratified by the year 1974 (the year in which the regulations on solvent use were promulgated), the SIR of the cohort of workers who were first employed prior to 1974 increased to 1.38 (95% confidence interval, 1.11–1.70). No such trend was discernible for workers employed after 1974. When 10 years of employment was considered, there was a further increase in the SIR for breast cancer, to 1.62. Those workers with breast cancer who were first employed prior to 1974 were employed at a younger age and for a longer period. Previous qualitative studies of interviews with the workers, corroborated by inspection records, showed a short-term high exposure to chlorinated alkanes and alkenes, particularly trichloroethylene before 1974. There were no similar findings on other types of cancer.

**Conclusion:**

Female workers with exposure to trichloroethylene and/or mixture of solvents, first employed prior to 1974, may have an excess risk of breast cancer.

## Background

The discovery of uncontrolled hazardous waste sites has emerged, in many countries, as an issue of major environmental and public health concern [1]. In 1994, a hazardous waste site, polluted by the dumping of solvents from a former factory, was discovered in Taoyuan, Taiwan; the factory was built in 1970, and was in operation for the manufacturing of electronic appliances up until 1992. This discovery subsequently emerged as a serious case of soil and groundwater contamination accompanied by suspected occupational cancer.

The factory had been inspected eight times by the Taiwanese government's inspection agency, with multiple violations of the regulations having been recorded. There were, for example, six separate citations amongst these violations for inadequate wind speed and/or capture velocity of the factory's ventilation devices [2]. The presence of tetrachloroethylene (PCE), 1,1-dichloroethylene, 1,1-dichloroethane, methylene chloride, trichloroethane (TCH), 1,1,2-trichloroethane, 1,2-dichloroethane and cis-1,2-dichloroethylene was subsequently detected in the soil and groundwater [3,4]. From a review of the literature at the International Agency for Research on Cancer (IARC), the US National Toxicology Program and the Integrated Risk Information System, we find an association has been reported between 1,1-dichloroethylene, 1,1-dichloroethane, methylene chloride, 1,2-dichloroethane and carbon tetrachloride, and the incidence of mammary gland adenocarcinomas in animals [5].

A prior study of risk assessment provided evidence on exposure to chlorinated alkanes and alkenes (CA) in community residents, accompanied by the increased risk of liver cancer [6,7], whilst a proportionate cancer morbidity ratio (PCMR) study found a significant increase in breast cancer (1.4 times) amongst workers in this company, as compared to workers in the weaving industry [8]. However, a subsequent standardized mortality ratio (SMR) study found no significant increase in cancer between 1990 and 1997 [9]. A further retrospective morbidity study, based on the same cohort, found a significant increase in the crude standardized incidence ratio for female breast cancer (SIR: 1.2, 95% CI: 1.0–1.4) [10]; however, the authors could find no significant dose-response related to duration of employment, and therefore make no conclusive inferences.

Nonetheless, a formal hypothesis linking breast cancer to solvents has been proposed by Canadian researchers, who found the persistent presence of many kinds of solvents in the breast milk of women which could potentially lead to an increase in the opportunity for initiating, or promoting cancer [11]. The period of adolescence, during which breast tissue undergoes substantial cellular proliferation, may diminish the time available for DNA repair [12]; thus, exposure to potential carcinogenic factors prior to such cellular differentiation may potentially increase the risk of cancer in later life [13]. Since an increase in mammary cancer has been found in female mice after chronic exposure to a mixture of CA [14], this retrospective cohort study was carried out based upon standardized incidence ratios (SIRs), in order to test the hypothesis of increased breast cancer amongst these female workers, with a 23-year follow-up period.

## Methods

A retrospective design was adopted for this study, incorporating secondary vital statistics data and employment records from the labor insurance database on a former electronics factory in Taiwan. The factory, which was located at latitude 24°N and longitude 121°E, covered an area of 36,000 km^2^. At the end of 2001, the total population in Taiwan was 22,092,387, comprising of 11,312,728 males and 10,779,659 females. The original cohort comprised of 64,000 female employees who had worked at the factory between 1973 and 1992. Three other workers with cancer were also excluded, because their diagnoses were established prior to the time of their first employment at the factory.

Following the further exclusion of 15 workers, each of whom had less than one full day of employment, we were left with a total of 63,982 workers for subsequent analysis. We obtained vital statistics data, reported by geographical location in Taiwan, from the Ministry of the Interior (MoI), within which both local and national vital statistics are centralized, along with census information. The Taiwan household registration program is designed to collate and supply demographic information, with every birth and death being ascertained by a formal certificate written and attested by a physician.

The coding of the sites of cancer was based upon the International Classification of Diseases for Oncology issued by the Department of Health, and the diagnoses of cancer for this cohort were determined by linking employee identification numbers for the entire cohort, from 1 January 1979 to 31 December 2001, with the data obtained from the Taiwan National Cancer Registry. The cancer registry is a population-based registry containing information on newly-diagnosed cancer patients in all hospitals in Taiwan with 50 beds or more. The registry is financially supported by a government agency, with registration fees being paid to the reporting hospitals on the basis of each case number reported.

The raw employment data on this cohort, provided by the Institute of Occupational Safety and Health and covered the period from 21 May 1973 to 31 December 1997, were retrieved from the database of the Bureau of Labor Insurance (BLI) of Taiwan. The overall duration of employment in this study was therefore accumulated from the beginning of the coverage of labor insurance to the date of the termination of employment.

Based upon knowledge amassed from our prior qualitative studies, for those subjects with incomplete BLI records, we made the following assumptions for the estimation of duration of employment. Where the date of first employment (and labor insurance) was missing, this was assumed to be the earliest age, i.e., 14 years; where the date of termination of employment was missing, this was assumed to be the end of factory operations in 1992. The Labor Insurance Act, promulgated by the government in 1958, subsequently came into effect in 1960. Thereafter, all Taiwanese citizens between the ages of 15 and 60 years were required to join the employment insurance program, as insured workers, either through their employer or through the establishment to which they belonged.

The latent periods of cancer in different organ systems are generally quite different. Although assuming minimal latency based upon a literature review may inadvertently increase the counting, within the denominators, of 'person-years at risk', we still made such choices in order to obtain conservative estimates. The latent periods of different cancers were: five years for cancer of thyroid [15], leukemia [16], 15 years for cancer of breast [17,18] and cervix uteri [19], and 10 years for the others [20].

Since workers in the early-1970s were exposed to high levels of solvents within a very short period of time, we stratified the durations of employment into less than, and more than, one year. 'Person-years at risk' for each individual worker were accrued after the assumed latent periods. The National Cancer Registry of Taiwan came into operation on 1 January 1979; thus, the collation of numbers of new cancer cases was based upon the earliest date of cancer occurrence, death, or the date when the person was last known to be alive and free of cancer on 31 December 2001. The number of cases was then stratified by age, calendar year, duration of employment, and the time of first employment.

Due to the lack of detailed information on exposure for each worker, we could only explore the dose-response relationship by stratifying the duration of employment into one month, and one, five, 10, 15 and 20 years. In order to account for the ambiguous nature of the latent periods, we conservatively apply the time since first employment as the time lag, thereby assuming that all workers became exposed once they were employed at the factory [21]. The short durations of exposure were decided by the extensive personal interviews from our qualitative study in 2003, which found short-term high exposure in female workers during the early-1970s [22].

No data on solvent exposure had been kept by the factory, and although we attempted to produce a reconstruction of such exposure, our dataset was too limited and crude to permit any possible linkage to individual workers. Following a serious outbreak of chemical hepatitis in another electronics factory in 1972 [23], the government issued strict regulations on the use of organic solvents within factories; these regulations were promulgated on 20 June 1974, followed by other amendments prior to 1990 (on 16 February 1976 and 21 November 1978) [24]. Under the current law in Taiwan, the use of CA is now strictly regulated in all workplaces.

The regulations in Taiwan cover trichloromethane (TCA), 1,1,2,2,-tetrachloroethane, tetra chloromethane, 1,2,-dichloroethylene, 1,2,-dichloroethane, carbon disulfide, TCE, PCE, methylene chloride, 1,1,1-trichloroethane and their mixtures. However, the factory inspection records on this company indicated that there were multiple violations of standards for ventilation devices, particularly in the early years [2]. We stratified the cohort into four groups to represent the cancer risks during different periods, according to the three promulgation and amendment dates noted above.

### Statistical Analysis

The PC Life Table Analysis System (LTAS) Version 1.0d was used to calculate the standardized incidence ratios (SIRs) for cancer. The system was developed by the Division of Surveillance, Hazards Evaluation and Field Studies (DSHEFS) of the US National Institute for Occupational Safety and Health (NIOSH), and provided for use by the public health community [25]. To be brief, the observed numbers of cancer cases amongst the cohort were compared with the expected numbers derived from the incidence rate amongst the general population in Taiwan during each calendar year. Expected numbers of cancer were calculated based on gender-, age-, and calendar time-specific incidence rates (five-year strata) of the Taiwan general population applied to the cohort's experience of person-years of follow-up in each specific stratum, and then summed up across different strata for different types of cancer. Then, the total numbers of observed cases were divided by the corresponding total expected number to obtain the SIR for a specific type of cancer, with a 95% confidence interval (CI) being also calculated under the assumption that the number of incidences had a Poisson distribution. To evaluate the potential impact of governmental regulation in 20 June 1974, the cohorts were divided into sub-cohorts of first employed before and after 1974, and estimated SIRs with 95% CI were also calculated for cancer types with case numbers larger than 50, namely, cancer of breast, cervix, thyroid, colon and rectum. Finally, t-tests were performed to compare the two sub-cohorts with breast cancer (employed before vs. after 1974) on three continuous outcomes, *i.e*., age at diagnosis, age at first employment, and length of employment.

This study was approved by the Ethics Review Board of the National Taiwan University College of Public Health before commencement.

## Results

A total of 63,982 female workers were recruited for this study, providing a total follow-up period of 1,403,824 person-years (without latent periods), as shown in Table 1. Within the whole sample 8,461 females first entered the factory during the high-exposure period, prior to 20 June 1974. More than half of them were less than 20 years of age at the time of their first employment, and 54 percent were employed for less than one year.

**Table 1:** Frequency distributions of demographic and cancer data on 63,982 female workers in electronics manufacturing

Variables	No.	%
First Employment		
Before 20 June 1974	8,461	13.2
20 June 1974–16 February 1976	6,778	10.6
16 February 1976–21 November 1978	12,624	19.7
After 21 November 1978	36,119	56.5

Age at first employment		
< 20 years old	36,370	56.8
≥ 20 years old	27,612	43.2

Duration of Employment		
< 1 month	13,496	21.1
1–11 months	20,969	32.9
1–4 years	11,504	18.0
5–9 years	4,655	7.3
≥ 10 years	13,358	20.9

Accumulated Person-years for Different Durations of Employment		
< 1 month	271,056	19.3
1–11 months	471,828	33.6
1–4 years	294,683	21.0
5–9 years	158,847	11.3
≥ 10 years	207,410	14.8

Cancer Sites		
Total (1979–2001)	1,572	100.0
Lip, Oral Cavity and Pharynx	59	3.8
Digestive Organs and Peritoneum	247	15.7
Respiratory System	60	3.8
Breast	385	24.5
Genital Organs	585	37.2
Urinary Organs	21	1.3
Other and Unspecified Sites	167	10.6
Lymphatic and Hematopoietic Tissue	48	3.1

After consideration for different latent periods of different types of cancer, no significant increases were found in the SIRs for any cancer sites except that of thyroid gland, as summarized in Table 2. Thyroid cancer risk was slightly but significantly lower than expected, and the SIR was 0.76 (95% CI: 0.58–0.98). When allowing 15 years of time lag following the time of first employment, this provided a total of 475,529 person-years of follow-up, with 286 cases of breast cancer. Further analysis, allowing 20 and 25 years of time lag after the time of first employment, also showed no significant increases for all types of cancer, with the exception of breast cancer. Where 20- and 25-year periods were allowed for time lag, then the respective SIRs for breast cancer were 1.18 (95% CI: 1.01–1.37) and 1.36 (95% CI: 1.04–1.75).

**Table 2:** Standardized incidence ratios (SIR) with 95% confidence interval (CI), by different types of cancer, 1979–2001

Cancer Sites	Lag Period (years)	Observed No. of Cancer Cases	Expected No. of Cancer Cases ^a^	SIR (95% CI)
Oral Cavity	10	10	13.5	0.74 (0.35–1.36)
Salivary glands	10	4	4.3	0.93 (0.25–2.38)
Nasopharynx	10	25	36.0	0.69 (0.45–1.03)
Esophagus	10	2	1.7	1.16 (0.14–4.20)
Stomach	10	42	47.6	0.88 (0.64–1.19)
Small intestine	10	3	3.0	1.01 (0.21–2.94)
Colon and rectum	10	98	89.3	1.10 (0.89–1.34)
Liver and intrahepatic bile ducts	10	36	45.5	0.79 (0.55–1.10)
Gall bladder and extrahepatic bile ducts	10	1	5.2	0.19 (0.005–1.07)
Pancreas	10	11	6.7	1.64 (0.82–2.94)
Peritoneum and other	10	6	3.4	1.77 (0.65–3.85)
Trachea, bronchus, and lung	10	46	49.9	0.92 (0.67–1.23)
Other parts of respiratory system	10	3	7.8	0.38 (0.08–1.13)
Breast	15	286	263.6	1.09 (0.96–1.22)
Cervix uteri	15	337	352.4	0.96 (0.86–1.06)
Other Parts of Uterus	15	25	25.9	0.96 (0.62–1.42)
Ovary, Fallopian Tube, and Broad Ligament	10	36	43.3	0.83 (0.58–1.15)
Other genital organs	10	7	7.0	0.99 (0.40–2.05)
Kidney and other Urinary Organs	10	15	13.6	1.10 (0.62–1.82)
Bladder	10	3	8.8	0.34 (0.07–1.00)
Skin	10	22	21.3	1.03 (0.65–1.56)
Brain	10	14	13.0	1.07 (0.59–1.80)
Other parts of nervous system	10	2	1.4	1.43 (0.17–5.17)
Thyroid gland	5	59	77.9	0.76 (0.58–0.98)
Bone	10	3	3.2	0.92 (0.19–2.70)
Connective tissue	10	11	8.4	1.31 (0.65–2.34)
Other and unspecified sites	10	24	28.4	0.84 (0.54–1.26)
Leukemia	5	23	29.5	0.78 (0.49–1.17)
All Sites	10	1,311	1,365.4	0.96 (0.91–1.01)

Notes:

^a^The expected number of cases amongst the total sample of 63,982 female workers was calculated based upon the age and calendar year-specific incidence rates of the general population.

Further cross-stratification was carried out for cancer of breast, thyroid, cervix, and colorectum based upon the duration of employment and the year of promulgation of the strict regulations on CA (20 June 1974), as summarized in Table 3. The significant increase in overall risk of breast cancer was found (SIR = 1.68; 95% CI: 1.11–2.42) if allowed for at least 10-year duration of employment. But increased risks in the strata of 10-year duration of employment were not found for cervical, colon and rectal cancer. No decreased risk of thyroid cancer was found for different duration of employment. A total of 90 cases of breast cancer were observed with an SIR of 1.38 (95% CI: 1.11–1.70) for the sub-cohort first employed prior to 1974. The SIR increased to 1.62 (95% CI: 1.02–2.42) if allowed more than 10-year duration of employment. There was no significant trend for increased risk with time since first employment. Furthermore, if workers employed for less than one year were excluded for fear of inadequate dose and/or induction time, the SIR became 1.36 (95% CI: 1.04–1.75). Among those female workers first employed before and after 1974, the SIRs for cervical, colorectal, and thyroid cancer were not significantly increased or decreased (Table 3).

**Table 3:** Comparison of observed to expected (O/E) numbers of cases and standardized incidence ratios (SIR) with 95% confidence interval (CI) for 4 different types of cancer amongst female workers, by calendar years and duration of employment

	Breast cancer		Cervical cancer		Colorectal cancer		Thyroid cancer	
	
Duration of Employment	O/E	SIR (95% CI)	O/E	SIR (95% CI)	O/E	SIR (95% CI)	O/E	SIR (95% CI)
Overall								
< 1 month	56/43.8	1.28 (0.97–1.66)	66/59.2	1.11 (0.86–1.42)	18/15.5	1.16 (0.69–1.84)	11/15.2	0.73 (0.36–1.30)
1–11 months	84/84.9	0.99 (0.79–1.22)	132/114.9	1.15 (0.96–1.36)	36/27.7	1.30 (0.91–1.80)	22/25.7	0.86 (0.54–1.30)
1–4 years	71/64.8	1.10 (0.86–1.38)	68/90.2	0.75 (0.59–0.96)	18/17.8	1.01 (0.60–1.60)	11/15.3	0.72 (0.36–1.29)
5–9 years	47/53.3	0.88 (0.65–1.17)	60/68.9	0.87 (0.66–1.12)	11/13.2	0.84 (0.42–1.50)	7/8.6	0.82 (0.33–1.69)
≥ 10 years	28/16.7	1.68 (1.11–2.42)	11/19.3	0.57 (0.28–1.02)	15/15.3	0.98 (0.55–1.62)	8/13.2	0.61 (0.26–1.20)
Total	286/263.6	1.09 (0.96–1.22)	337/352.4	0.96 (0.86–1.06)	98/89.3	1.10 (0.89–1.34)	59/77.9	0.76 (0.58–0.98)

Before 20 June 1974								
< 1 month	11/5.6	1.97 (0.98–3.52)	5/7.4	0.68 (0.22–1.58)	1/1.8	0.57 (0.01–3.15)	0/1.1	-
1–11 months	19/15.6	1.22 (0.73–1.90)	26/20.8	1.25 (0.82–1.83)	5/4.9	1.02 (0.33–2.39)	2/2.9	0.68 (0.08–2.47)
1–4 years	17/12.3	1.38 (0.81–2.22)	16/17.3	0.93 (0.53–1.50)	2/3.6	0.56 (0.06–2.02)	2/1.9	1.03 (0.12–3.71)
5–9 years	20/17.5	1.14 (0.70–1.76)	14/23.3	0.60 (0.33–1.01)	7/4.0	1.73 (0.69–3.57)	0/2.7	-
≥ 10 years	23/14.2	1.62 (1.02–2.42)	11/16.5	0.67 (0.33–1.19)	6/6.3	0.96 (0.35–2.08)	1/3.6	0.28 (0.004–1.54)
Total	90/65.2	1.38 (1.11–1.70)	72/85.2	0.84 (0.66–1.06)	21/20.5	1.02 (0.63–1.56)	5/12.2	0.41 (0.13–0.95)

After 20 June 1974								
< 1 month	45/38.2	1.18 (0.86–1.57)	61/51.8	1.18 (0.90–1.51)	17/13.7	1.24 (0.72–1.99)	11/14.1	0.78 (0.39–1.39)
1–11 months	65/69.3	0.94 (0.72–1.20)	106/94.1	1.13 (0.92–1.36)	31/22.8	1.36 (0.92–1.93)	20/22.8	0.88 (0.54–1.36)
1–4 years	54/52.5	1.03 (0.77–1.34)	52/72.9	0.71 (0.53–0.94)	16/14.2	1.13 (0.64–1.83)	9/13.3	0.68 (0.31–1.28)
5–9 years	27/35.8	0.75 (0.50–1.10)	46/45.7	1.01 (0.74–1.34)	4/9.1	0.44 (0.12–1.12)	7/5.9	1.20 (0.48–2.46)
≥ 10 years	5/2.5	2.03 (0.65–4.74)	0/2.8	-	9/9.0	1.00 (0.46–1.90)	7/9.6	0.73 (0.29–1.51)
Total	196/198.3	0.99 (0.85–1.14)	265/267.2	0.99 (0.88–1.12)	77/68.8	1.12 (0.88–1.40)	54/65.6	0.82 (0.62–1.07)

Of the 90 cases of breast cancer, 11 were employed for less than one month, with an increased SIR of 1.97 (95% CI: 0.98–3.52). Workers with breast cancer who were first employed prior to 1974 (19.6 ± 6.1 years old) were younger at the time of their first employment and were employed for longer periods; furthermore, at the time of their diagnosis, these workers were older than those who had first been employed after 1974, as shown in Table 4.

**Table 4:** Comparison of frequency distributions between two breast cancer groups, by date of first employment

	Date of First Employment					
	
	Before 20 June 1974 (n = 90)			After 20 June 1974 (n = 196)		
	
	Mean	SD	Range (years)	Mean	SD	Range (years)
Age at Diagnosis	44.3	7.1	31–70	42.6	6.9	30–63
Age at First Employment*	19.6	6.1	14–46	22.4	6.3	14–43
Length of Employment*	7.8	7.5	0.005–25	4.8	6.5	0.008–18.5

Note: * indicates p < 0.001 by Student t-test

## Discussion

Although we found an increased risk of breast cancer amongst young female workers first employed prior to 1974 (as shown in Table 3), it does not necessarily follow that such an association is causal, since our data did not allow for the control of potential confounders, including exposure to ionizing radiation [26-28], smoking [29], alcohol consumption [30,31], menopause after the age of 55 [32], low parity [33-38], late first pregnancy [39-41], and a positive family history [42-44]. Thus, careful scrutiny of the findings should be undertaken prior to drawing any conclusions on the association.

**Figure 1:**
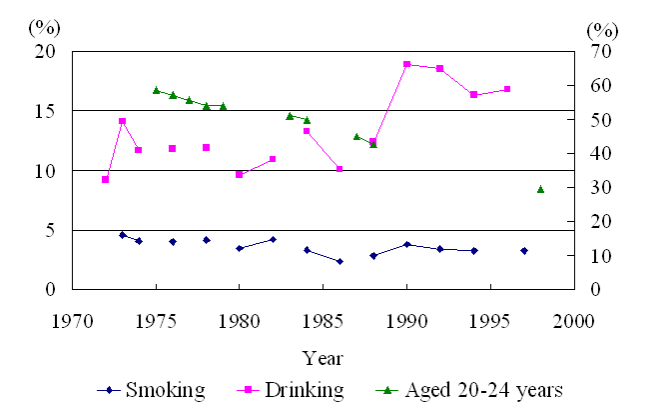
Prevalence rates for smoking and drinking in female adults (percentage of left-hand side), and the proportion of births of first offspring to mothers aged 20–24 years (percentage of right-hand side) in Taiwan, 1972–2001 [45-47].

Following promulgation, on 16 April 1974, Article 12 of the Labor Safety and Health Act in Taiwan, required every worker to undergo a pre-employment physical examination, which included a chest roentgenography. Female workers first employed in Taiwan prior to 1975 were therefore less likely to be exposed to ionizing radiation than those whose employment began later; thus, it seemed unlikely that the hypothesis of increased ionizing radiation was a potential confounder within this association. Similarly, according to the regular survey data undertaken by the Tobacco and Wine Monopoly Bureau, the smoking and alcohol consumption rates of adult women were relatively low in the early-1970s; there has, however, been an increasing trend over the last three decades for alcohol but not for smoking, as illustrated in Figure 1[45,46]. Therefore, it also seems unlikely that either smoking or alcohol consumption were potential confounders in this study.

Of the 286 cases of breast cancer in females, one was below 30 age group, 101 were in the 30–39 age group, 144 were in the 40–49 age group and the remaining 40 cases were in the above 50 age group. Thus, menopause after the age of 50 cannot explain the increased risk. Moreover, since the age of bearing a first child has tended to shift over the past three decades, from 20–24 years to 25–29 years, or even into the early-thirties (Figure 1), the likelihood of a late first pregnancy for women first employed prior to 1974 was also lower [47]. It therefore appears that family history was the only factor we were unable to rule out.

**Figure 2:**
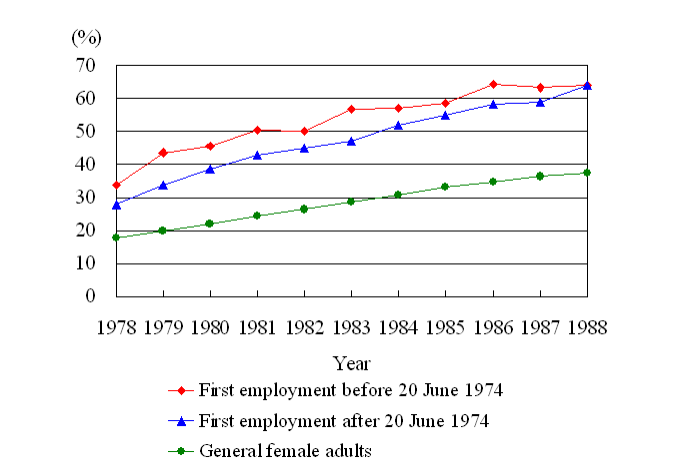
Proportion of senior high and vocational schools or above of 47,348 female workers in comparison with general female adults in Taiwan, 1978–1988 [49].

Historically, a higher socioeconomic status (SES) has been reported to be associated with increased breast cancer verified by a number of epidemiological studies, but few previous studies could adjust for all relevant potential confounding factors. Braaten et al. suggested that this positive gradient in risk for breast cancer can be fully explained by established breast cancer risk factors [48]. Although there was no information on level of education from the BLI database of original cohort, we still retrieved relevant educational data of 47,348 workers (74%) by linking to the Taiwan Birth Registration Database that began in 1978 [49]. The proportions of senior high and senior vocational schools for female workers were significantly higher than general female adults, as summarized in Figure 2. Based on the employees' earnings survey of Directorate General of Budget, Accounting and Statistics, the income of female workers in electronics manufacturing were above the average in the early 1970s [50], which was raised from 124 to 486 US dollars during the period between 1978 and 1988. As this factory is invested and owned by multinational enterprise, the wages were even higher than average electronics [22]. On the contrary, female workers in this factory were in a higher socioeconomic class, which might be explanatory to the decreased morbidity due to thyroid cancer, as was also found by Levi et al [51]. And there might still be possibility of residual confounding by SES for increased risk of breast cancer.

Since this study used the same dataset of cancer registry to link both the exposed cohort and the comparison cohort, and since it ensured that all cancer cases were ascertained, there was very good comparability in the collection of information; thus, any selection bias was minimal, or indeed negligible. Moreover, although we do not have the actual exposure data, random misclassification generally leads toward null [52]. The assumptions of the first and last dates of employment for missing values would also generally lead to an underestimation of SIR.

Throughout its period of operation, a wide range of solvents were used within this factory [53,54], including isopropyl alcohol (IPA), acetone, methyl ethyl ketone (MEK), cleaner, cobehn spray (trichloromethane, TCA) and 1,1,1-trichloroethane, and trichloroethylene (TCE). Furthermore, TCE was once used extensively for degreasing and cleaning [22,53], with our prior qualitative study indicating that many female workers had used the abovementioned CA on a daily basis prior to 1974, for cleaning dirt off clothes, wiping job utensils and tabletops, and even for washing their hands [22].

The factory's labor inspection records also indicated that: (i) the wind speed of the factory ventilation system was too slow to exhaust the fumes; (ii) workers were not educated fully about solvent toxicity; and (iii) only cotton gloves were provided by the company for protection against skin contact [2]. Furthermore, as reported in Table 5, TCE was no longer cited in the records after 1974. Thus, we suspect that exposure was generally quite heavy, with the increased SIR in workers employed for more than 10 year also corroborating the biological plausibility.

**Table 5:** Solvents appearing in the company's monthly magazines and labor inspection records between August 1973 and May 1991, sorted by priority dates

Period of Use	Citations of Solvents in Use
Before 20 June 1974	1. Trichloroethylene [53] (Aug/Sep 1973)

After 20 June 1974	1. Isopropyl alcohol [2,54]
	2. Acetone [2,54]
	3. Methyl ethyl ketone [2,54]
	4. Trichloromethane [54]
	5. Methylene chloride [2]
	6. Toluene [2]
	7. Petroleum naphtha [2]
	8. N-hexane [2]
	9. Ethyl acetate [2]
	10. Methyl alcohol [2]
	11. 1,2, – dichloroethylene [2]
	12. 1,1,1, – trichloroethane [2]
	13. 1,2, – dichloroethane [2]
	14. 1,1,2, – trichloroethane [2]
	15. Tetrachloroethylene [2]

An extensive review suggests an association of TCE exposure with kidney cancer, liver cancer, and non-Hodgkin's lymphoma as well as for cervical cancer, Hodgkin's disease, and multiple myeloma [55]. All of above cancers were analyzed in our study but no increase of SIR has been found. The analysis is also limited by the lack of detailed exposure information.

After stratification by the year of first employment, the SIR for breast cancer increased from 1.09 to 1.38 (Table 3); if allowing 10 years of employment, this increased further to 1.62 (95% CI: 1.02–2.42). Since the cancer registry database was only available for the period up to 2001, any workers whose first employment was after 1986 were not eligible for inclusion within the cohort if a latent period of 15 years was taken into consideration. Thus, we tentatively conclude that an association does exist for the period prior to 1974, when CA was in use, and that there is a need for continued follow-up to further examine this association.

## Conclusion

Due to the lack of detailed information on the early-1970s, we can make only limited inferences with regard to overall exposure. Based upon our in-depth review of formal inspection records [2], and the monthly magazines published by the company [53,54], we find that the only organic solvent under strict regulation which no longer appeared after 1974, was TCE (Table 5). We therefore suspect that TCE, and/or its mixtures, may be the most likely agent responsible for our findings. However, our results should be interpreted with caution since it is clear that co-exposure to other solvents did occur amongst our subjects.

In summary, there was a significantly increased risk of breast cancer amongst female workers within this factory prior to the introduction of strict regulations on the use of CA in all workplaces. Such risks were more prominent amongst female workers entering the workforce prior to 1974. However, our inference is limited by the lack of individual information on both the intensity of occupational exposure and family history.

## Competing interests

The author(s) declare that they have no competing interests.

## Authors' contributions

TIS and JDW designed the study and collected all data, and were fully responsible for the interpretation of all results, and JDW supervised the entire study. TIS developed the protocol for this study with assistance from PCC, LJHL and YPL, and also performed the statistical analysis with assistance from PCC and GYH. All authors contributed drafting the manuscript.

## Pre-publication history

The pre-publication history for this paper can be accessed here:


